# Adjacent single-stranded regions mediate processing of tRNA precursors by RNase E direct entry

**DOI:** 10.1093/nar/gkt1403

**Published:** 2014-01-21

**Authors:** Louise Kime, Justin E. Clarke, David Romero A., Jane A. Grasby, Kenneth J. McDowall

**Affiliations:** ^1^Astbury Centre for Structural Molecular Biology, School of Molecular and Cellular Biology, University of Leeds, Leeds LS2 9JT, UK and ^2^Department of Chemistry, University of Sheffield, Sheffield S3 7HF, UK

## Abstract

The RNase E family is renowned for being central to the processing and decay of all types of RNA in many species of bacteria, as well as providing the first examples of endonucleases that can recognize 5′-monophosphorylated ends thereby increasing the efficiency of cleavage. However, there is increasing evidence that some transcripts can be cleaved efficiently by *Escherichia coli* RNase E via direct entry, i.e. in the absence of the recognition of a 5′-monophosphorylated end. Here, we provide biochemical evidence that direct entry is central to the processing of transfer RNA (tRNA) in *E. coli*, one of the core functions of RNase E, and show that it is mediated by specific unpaired regions that are adjacent, but not contiguous to segments cleaved by RNase E. In addition, we find that direct entry at a site on the 5′ side of a tRNA precursor triggers a series of 5′-monophosphate-dependent cleavages. Consistent with a major role for direct entry in tRNA processing, we provide additional evidence that a 5′-monophosphate is not required to activate the catalysis step in cleavage. Other examples of tRNA precursors processed via direct entry are also provided. Thus, it appears increasingly that direct entry by RNase E has a major role in bacterial RNA metabolism.

## INTRODUCTION

In *Escherichia coli*, the rapid degradation of many, if not most transcripts, including messenger RNAs (mRNAs) targeted by antisense RNAs, is dependent on RNase E [for recent reviews, see (1,2)], a single strand-specific endonuclease that also has a key role in the processing of precursors of ribosomal RNA (3–5) and transfer RNA (tRNA) (6,7), as well as several small non–protein-coding RNAs (8,9). Reflecting its central role in RNA processing and degradation, RNase E is essential for *E. coli* viability (8–11). Its contribution to RNA metabolism has been studied extensively using two temperature-sensitive *rne* mutations (10,11) that have been mapped to the catalytic domain (12,13). Homologues of RNase E are found in most subdivisions of bacteria and within plant plastids (14,15). *E. coli* contains a paralogue, RNase G, that cooperates with RNase E in the maturation of 16S ribosomal RNA (16,17) and has been shown to be required for the normal degradation of several mRNAs (18), some of which have been characterized (19–21).

*Escherichia coli* RNase E is integral to the RNA degradosome, a macromolecular complex located on the inner surface of the cytoplasmic membrane [for reviews, see (1,2,22)]. The other ribonuclease within this complex is polynucleotide phosphorylase, a 3′–5′ exonuclease [for review, see (23)]. The endonucleolytic activity of RNase E is conferred by its N-terminal half (NTH) (13,24), which self-associates to form a tetramer (25) via the dimerization of a dimer: each dimeric unit forms two symmetrical active sites set within single-stranded-RNA-binding channels (26). The sites of interaction with the other main components of the degradosome, along with ancillary RNA-binding sites, are contained within the C-terminal half of RNase E (13,27,28), which although required for efficient growth is not essential for cell viability (29,30).

Located adjacent to each of the four equivalent active sites in RNase E is a pocket that can bind a 5′-monophosphorylated end, i.e. contacts are made with the first few nucleotides and the actual 5′-monophosphate group (26). Moreover, RNase E has been shown *in vitro* to cleave more efficiently the 5′-monophosphorylated versions of certain oligonucleotide substrates and transcripts relative to counterparts with a hydroxyl or triphosphate, respectively, at their 5′ end (21,24,31–34). Thus, a 5′-monophosphate can ‘tag’ some RNAs for efficient cleavage by RNase E. Furthermore, as RNase E generates downstream products with a 5′ monophosphate (5), it has been proposed that these products may be cleaved preferentially, triggering a cascade of cleavages in the 5′–3′ direction (34).

Recently, *E. coli* and other bacteria have been found to contain a 5′ pyrophosphatase (now called RppH) that converts the 5′ group of primary transcripts from a tri- to monophosphate (35). RppH is not essential; however, its genetic inactivation results in the stabilization of a significant proportion of *E. coli* mRNAs (36). Thus, pyrophosphate removal by RppH appears to accelerate the degradation of many transcripts (37). Stem-loops (or paired nucleotides) at the 5′ end of transcripts reduce the efficiency of pyrophosphate removal by RppH (36) and 5′-end sensing by RNase E (34), thereby protecting some transcripts against rapid degradation *in vivo* (38–42). An important role for events at the 5′ end in controlling RNA degradation is further supported by the finding that circularization of an mRNA increased its half-life *in vivo* (43).

Initially it was thought that 5′-monophosphoryated ends might stimulate cleavage by *E. coli* RNase E and RNase G by enhancing primarily the turnover number (44), perhaps by triggering an allosteric switch in enzyme conformation (26,45). However, it was subsequently shown that RNase G has a much higher affinity for 5′-monophosphorylated oligonucleotide substrates (21), and that RNase E could cleave 5′-hydroxylated oligonucleotides as efficiently as 5′-monophosphorylated substrates provided the former were bonded to present a substrate with multiple single-stranded regions (46). Thus, the absence of 5′-monophosphate binding might not present an intrinsic barrier to catalysis, provided the substrate can be bound with sufficient affinity. Moreover, the tetrameric structure of RNase E means that it has the capacity to achieve the latter by contacting simultaneously single-stranded segments in addition to the one in which cleavage occurs. The apparent simplicity of these requirements for 5′-monophosphate-independent cleavage raises the possibility, which remains to be adequately explored, that this mode of cleavage is used widely to accelerate mRNA degradation. Direct entry could explain at least in part why the normal rapid degradation of only a proportion of the mRNAs in *E. coli* is highly dependent on 5′ pyrophosphate removal by RppH (46).

Central to more recent studies of the RNase E family is the mutation of residues that contact 5′-monophosphorylated ends (21,31,32,46); Arg 169 and Thr 170, which provide a horseshoe of hydrogen bond donors that engage the monophosphate group, and Val 128, which provides a hydrophobic side chain that interacts with the aromatic ring of the terminal base (26). Here, we used the T170V mutation of *E. coli* RNase E, which reduces the efficiency of cleavage of 5′-monophosphorylated oligonucleotides (46), to examine the substrate requirements for tRNA processing (47). We were drawn to study these substrates not only because their processing represents one of the main activities of RNase E (6,7) in *E. coli* and other bacteria (48), but because the localized folding that produces tRNAs limits the formation of alternative secondary structures within the precursor (and derivatives) that can complicate the analysis of RNA: protein interactions. We focused on the processing of the polycistronic *argX-hisR-leuT-proM* precursor, as it has been the subject of *in vivo* studies by others (6,7), including a recent study that concluded its processing was not dependent on the 5′ sensor of RNase E (49). Our study confirms that direct entry is central to the processing of tRNA in *E. coli* and provides the first biochemical evidence for natural transcripts that direct entry is mediated by specific unpaired regions that are adjacent to, but not contiguous with, segments cleaved by RNase E. In addition, we find evidence that direct entry at a site on the 5′ side of the precursor triggers a series of 5′-monophosphate-dependent cleavages. Consistent with a major role for direct entry in tRNA processing, we show also that, contrary to a report by others (32), a 5′-monophosphate is not required to ‘activate’ the catalytic step (44).

## MATERIALS AND METHODS

### Synthesis of RNA transcripts

Transcripts were synthesized *in vitro* using T7 RNA polymerase and polymerase chain reaction-generated templates and purified as described previously (46,50). The sequences of the primers used to generate templates are given in Table 1.

**Table 1.** The sequences of primers used to generate templates for *in vitro* transcription

Transcript	Primer	Primer sequence (5′–3′)
*argX-hisR-leuT-proM* precursor	FWD	ATCCTAATACGACTCACTATAGGGAACGGCGCTAAGCGCCCG
	RVS	AAAAAACCCCGCCGAAGCGG
5′ *hisR* to 3′	FWD	ATCCTAATACGACTCACTATAGGGGGTGGCTATAGCTCAGTTGG
	RVS	AAAAAACCCCGCCGAAGCGG
5′ *hisR* to 3′ *proM*	FWD	ATCCTAATACGACTCACTATAGGGGGTGGCTATAGCTCAGTTGG
	RVS	TGGTCGGCGAGAGAGGAT
5′ *leuT* to 3′	FWD	ATCCTAATACGACTCACTATAGGGGCGAAGGTGGCGGAATTGGT
	RVS	AAAAAACCCCGCCGAAGCGG
5′–3′ *leuT*	FWD	ATCCTAATACGACTCACTATAGGGAACGGCGCTAAGCGCCCG
	RVS	TGGTGCGAGGGGGGG
5′–3′ *hisR*	FWD	ATCCTAATACGACTCACTATAGGGAACGGCGCTAAGCGCCCG
	RVS	TGGGGTGGCTAATGGGATT
5′ *argX* to 3′ *hisR*	FWD	ATCCTAATACGACTCACTATAGGGGCGCCCGTAGCTCAGCTG
	RVS	TGGGGTGGCTAATGGGATT
5′–3′ *proM*	FWD	ATCCTAATACGACTCACTATAGGGAACGGCGCTAAGCGCCCG
	RVS	TGGTCGGCGAGAGAGGAT
*metT-leuW-glnUW-metU-glnVX* precursor	FWD	ATCCTAATACGACTCACTATAGGGCGCAACGCCGATAAGGTA
	RVS	ATTGAATGAACGCAGAAAAGC
*glyVXY* precursor	FWD	ATCCTAATACGACTCACTATAGGGCCGTAACGACGCAGAAATG
	RVS	GCGTCGCTGTGGATATTTTATT

The T7 polymerase promoter encoded in each of the forward primers is underlined.

To generate transcripts with 5′-monophosphorylated ends, the RNA was incubated with tobacco acid pyrophosphatase (TAP; Epicentre® Biotechnologies) in a ratio of 25 U TAP: 8 µg RNA in a 50 µl reaction using buffer provided by the vendor at 37°C for 2 h. The RNA was extracted with phenol-chloroform and precipitated with ethanol as described previously (50). The 5′-phosphorylation status of transcripts was determined using a 5′–3′ exonuclease specific for 5′-monophosphorylated RNA. The reaction (20 µl) contained 300 ng RNA and 0.1 U Terminator™ exonuclease (TEX; Epicentre® Biotechnologies) in buffer B provided by the vendor. After incubation for 30 min at 42°C the RNA was extracted with phenol-chloroform and precipitated with ethanol and analysed by denaturing polyacrylamide gel electrophoresis.

### Annealing of complementary DNA oligonucleotides to *in vitro* transcribed RNA

The sequences of oligonucleotide primers used to anneal to RNA transcripts are given in Table 2.

**Table 2.** The sequences of primers used for annealing to complementary regions in RNA transcripts

Primer name	Primer sequence (5′–3′)
Block-E1	CTACAAATCTTGTTACGCGGTATTA
*argX-hisR* 5′ intergenic region	CAGCTCAAGCGCCGGGACTA
*argX-hisR* centre intergenic region	TATTACTACCACCGCAGC
Block-E2	TTGTCACAACTTCTAATAA
Block-E3	TTTTAGTTCAATTCTTTAAAGTCG
Block-E4	AATACTGCTTTTTGAATTTTTAG

To anneal, the RNA in water was heated to 95°C for 3 min. Following addition of complementary oligonucleotide, the reaction was incubated at 65°C for 5 min, 35°C for 5 min and then placed on ice. Specific oligonucleotide binding was confirmed by treatment with RNase H, which specifically cleaves the RNA in RNA–DNA hybrids. RNA-oligonucleotide (2 pmol) was incubated at 37°C for 1 h with 2.5 U RNase H in buffer provided by the vendor (Fermentas Life Sciences). The reaction products were analysed by denaturing polyacrylamide gel electrophoresis.

### Purification of NTH-RNase E and discontinuous cleavage assays

Recombinant N-terminal histidine-tagged polypeptides corresponding to the NTH of RNase E (residues 1–529) with wild-type or mutant sequences were purified as described previously (46). The cleavage assays were performed also as described previously (46). The LU13 oligonucleotide substrates labelled with fluorescein at the 3′-end were synthesized and purified by Eurogentec (UK). The sequence of LU13 was 5′-GAGACAGU↓AUUUG (arrow indicates site of cleavage). To estimate *k*_cat_ and *K*_M_ values of the cleavage of 5′-hydroxylated LU13, initial rates were calculated from time points within the linear phase of the reaction. These rates were then fitted to the Michaelis–Menten function as shown in Equation (1),
(1)
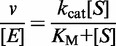

Where *v* is the initial rate normalized for [*E*] (the total enzyme concentration), [*S*] is the initial substrate concentration, *k*_cat_ is the enzyme turnover number and *K*_M_ is the Michaelis constant.

## RESULTS

### A major role for direct entry

**Figure 1.**
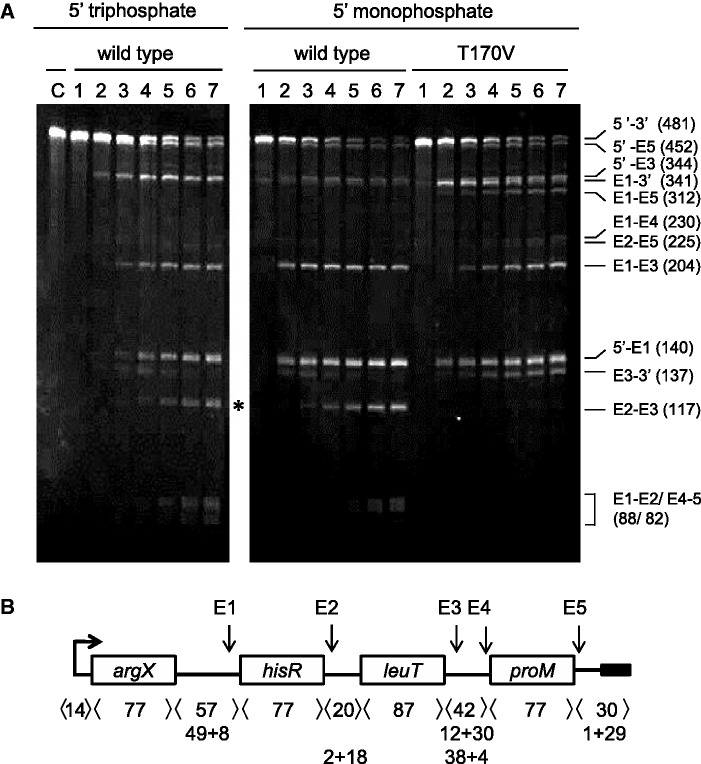
The role of 5′ sensing in the cleavage of the polycistronic *argX-hisR-leuT-proM* precursor by RNase E. (**A**) The effect of 5′ phosphorylation and sensing on cleavage by the NTH of RNase E. The precursor was generated by *in vitro* transcription (see ‘Materials and Methods’ section, for further details). The 5′-monophosphorylated version was generated using TAP (51). The reaction conditions and preparations of both wild-type NTH-RNase E and the T170V mutant were as described previously (46). Products were analysed using denaturing gel electrophoresis (46). An asterisk indicates a species referred to in the text. The enzyme monomer and initial substrate concentrations at the start of each reaction were 5 and 180 nM, respectively. The RNA was stained using ethidium bromide. Lanes 1–7 contain samples taken 0, 5, 15, 30, 60, 120 and 180 min after mixing substrate and enzyme. Lane C contains substrate incubated without enzyme for 180 min. The identities of the bands are indicated on the right of the panel (see text for details). Their sizes in nucleotides are given in parentheses. (**B**) Schematic diagram showing the positions of sites of cleavage by RNase E (6) (our unpublished data). The three tracks of values below the tRNA positions indicate the length of each segment (nucleotides), the sizes of segments following cleavage at E1, E3 and E5 and following cleavage at E2 and E4, respectively.

The starting point for our analysis of tRNA processing was the cleavage of a 5′-monophosphorylated form of the *argX-hisR-leuT-proM* precursor by NTH-RNase E. This was then compared against the cleavage of the same substrate by the T170V mutant and a 5′-triphosphorylated form by NTH-RNase E to assess the contribution of direct entry (Figure 1, panel A). Reaction conditions were used that had been shown previously to facilitate only limited cleavage of 5′-triphosphorylated versions of well-characterized 5′-monophosphate-dependent substrates (46,50). We found that the efficiency of the initial cleavages, as determined by the reduction in abundance of full-length precursor, was not decreased substantially when the substrate was incubated with the T170V mutant, or when its 5′ end was triphosphorylated (Figure 1, panel A). These results confirmed that direct entry has a substantial role in the processing of the *argX-hisR-leuT-proM* precursor. However, 5′-end-dependent cleavage does contribute, as evidenced most clearly by the accumulation of a shorter product (marked by an asterisk) following incubation with wild-type NTH-RNase E, but not its T170V equivalent. Before undertaking the comparisons described above, we had established that the cleavage products produced by the NTH of RNase E were the same as those produced by the RNA degradosome under conditions in which PNPase was not active (data not shown). Others have also found that NTH of RNase E is sufficient to direct all of the cleavages produced by the degradosome (31). We chose to base our analysis of 5′ sensing on the NTH-RNase E rather than the degradosome, as we have so far been unable to purify degradosome preparations that incorporate RNase E with mutations in its 5′ sensor.

Next, the identity of each of the cleavage products was determined by tagging the 5′ and 3′ ends with extended sequences (Supplementary Figures S1 and S2), and comparing the electrophoretic mobility of products against RNA size markers (Supplementary Figure S3), truncating the substrate, and using complementary oligonucleotides to block RNase E cleavage at sites mapped previously by others (6). This revealed that the major sites of direct-entry cleavage by RNase E occurred at E1, E3 and E5 with additional 5′-monophosphate-dependent steps requiring cleavage at E2: cleavage at E4 was also detected under particular conditions (Figure 1, panel B). E5 was previously uncharacterized but likely serves to remove the transcription terminator on the 3′ side of *proM* (52). RNase E-dependent cleavage was detected *in vivo* at E5, as well as E1 to E4, by comparing the abundance of 5′ ends in an *rne-1^ts^* strain and its congenic wild-type partner at the non-permissive temperature (our unpublished RNA-seq data). Much of the mapping just outlined earlier in the text is presented later in the text as part of our analysis of individual sites of cleavage. E2, E3 and E5 are located within 15 nt of the 3′ end of the corresponding tRNAs, whereas E1 and E4 are more distal (Figure 1, panel B). All the cleavages occurred within segments that are single stranded and rich in A and/or U nucleotides (6,48). These are characteristics typical of sites of RNase E cleavage (53,54). The sequence of the *argX-hisR-leuT-proM* precursor annotated to show the precise positions of all the RNase E sites and the sequences blocked by complementary oligonucleotides is provided (Supplementary Figure S4).

### Requirements for direct-entry cleavage at E3 and E5

**Figure 2.**
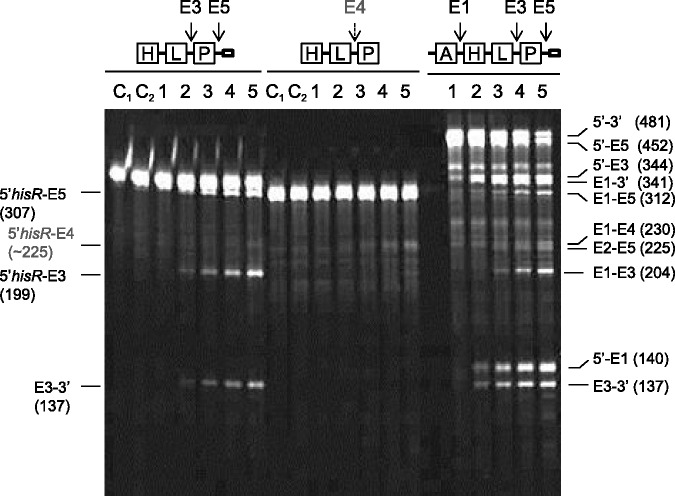
The requirements for direct-entry cleavage at E3. Truncated versions of the *argX-hisR-leuT-proM* precursor were generated by shortening the template used for *in vitro* transcription. All of the transcripts had 5′-triphosphorylated ends and were incubated with T170V. The boundaries of each of the transcripts and the positions at which they were cleaved are shown schematically at the top of each panel. The cleavage of the transcripts was assayed as Figure 1. The enzyme and initial substrate concentrations at the start of each reaction were 7 and 250 nM, respectively. Lanes 1–5 contain samples taken 0, 5, 15, 30 and 60 min after mixing substrate and enzyme. Lanes C_1_ and C_2_ correspond to substrate incubated without enzyme for 0 and 60 min. The RNA was stained using SYBR® Gold stain (Life Technologies). The identities and sizes of the species produced by cleavage of the precursor starting at the 5′ end of *hisR* tRNA are indicated on the left of the panel. The identities and sizes of the products of cleaving full-length transcript are indicated on the right of the panel, as Figure 1.

**Figure 3.**
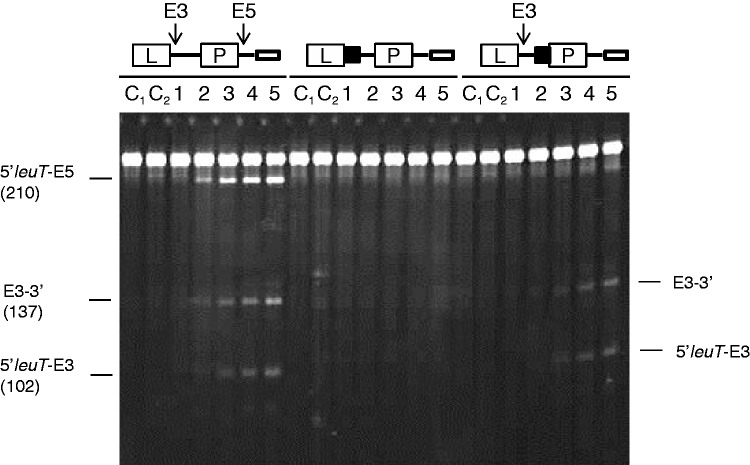
The requirements for direct-entry cleavage at E5. The 5′-triphosphorylated substrates incubated with T170V are shown schematically at the top of the panel. Closed black boxes indicate the binding sites to two complementary oligonucleotides used to block cleavage. Only sites at which cleavage occurs are indicated. Their precise locations are shown in Supplementary Figure S4. The transcript in each case started at the 5′ end of *leuT* tRNA. As Figure 2, lanes 1–5 contain samples taken 0, 5, 15, 30 and 60 min after mixing substrate and enzyme, whereas lanes C_1_ and C_2_ correspond to substrate incubated without enzyme for 0 and 60 min. Enzyme and initial substrate concentration and staining of products as Figure 2. The labelling on the right and left indicates the species produced in the presence and absence of oligonucleotide binding to a segment encompassing E4.

To study the requirement for direct-entry cleavage at E3 and E5, without the complication of cleavage at E1, the segment of the precursor upstream of *hisR* was removed. Incubation of the resulting 5′-triphosphorylated transcript with T170V produced three major detectable products in what appears to be stoichiometric amounts (after taking into account the size-dependent differences in staining); 5′ *hisR* to E5 (307 nt), 5′ *hisR* to E3 (199 nt) and E3-3′ (137 nt) (Figure 2). E5 to 3′ (29 nt) was too small to be detected. A much weaker band that probably corresponds to 5′ *hisR* to E4 (225 nt) was also detected. Interestingly, no E3 to E5 product (108 nt) was detected, even after extending the incubation with a higher concentration of T170V (data not shown). The above indicated that T170V can cleave efficiently at either E3 or E5 by direct entry, but not both. More remarkably, removal of the segment downstream of *proM*, which contains the E5 site, was found to block completely cleavage at E3: only the weak band assigned to 5′ *hisR* to E4 was detected. The E3 site remained single stranded as judged by the ability of a complementary oligonucleotide described later in the text to direct cleavage by RNase H (data not shown). This was the first indication that direct entry might require recognition of an unpaired region that is adjacent, but not contiguous to a segment in which RNase E cleavage can occur. Repeating the study with a substrate truncated upstream of *leuT* produced identical findings and confirmed the identity of the cleavage products (data not shown, also Figure 3).

The finding that T170V can cleave a 5′-triphosphorylated transcript efficiently at E3 or E5, but not both, suggested a model in which RNase E interacts with the 3′ half of the *argX-hisR-leuT-proM* precursor via simultaneous contact with single-stranded regions encompassing the E3 and E5 sites and that subsequent cleavage at E3 or E5 reduces the affinity of the interaction such that cleavage at the other cannot occur via this route. As predicted by this model, the binding of an oligonucleotide complementary to the E3 site completely blocked cleavage at E5, as well as at E3 (Figure 3). This was shown using a substrate with the region upstream of *leuT* removed. It is clear that the 5′ *leuT* to E5 (210 nt) species was no longer produced. Cleavage at E5 was also blocked by the binding of an oligonucleotide complementary to the E4 site, which is located downstream of E3 within the intergenic region of *leuT* and *proM*. This did not, however, block cleavage at E3; both the E3 to 3′ and 5′ *leuT* to E3 species were produced. Thus, the binding events that normally lead to cleavage at E3 or E5 are not identical; cleavage at E5 appears to require an additional or extended contact not required for cleavage at E3. Nevertheless, as found for E3, cleavage at E5 requires an unpaired region that is adjacent, but not contiguous to the site of E5 cleavage. Specific annealing of complementary oligonucleotides to the substrate was confirmed by RNase H digestion (Supplementary Figure S5).

### Requirements for direct-entry cleavage at E1

**Figure 4.**
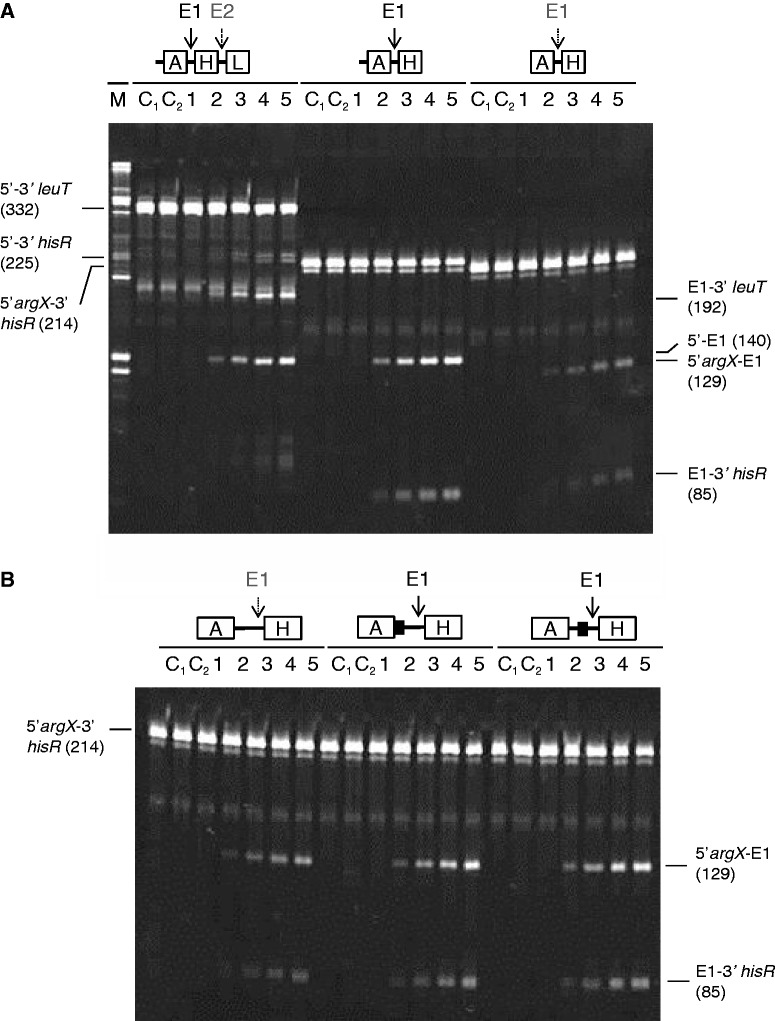
The requirements for direct-entry cleavage at E1. (**A**) Identifying the minimum substrate. The triphosphorylated substrates were incubated with T170V, and the positions of the resulting cleavages are shown schematically at the top of this panel. As Figure 2, lanes 1–5 contain samples taken 0, 5, 15, 30 and 60 min after mixing substrate and enzyme, whereas lanes C_1_ and C_2_ correspond to substrate incubated without enzyme for 0 and 60 min. Lane M contains the products from cleaving full-length precursor: these are provided as size markers. The labelling on the right indicates the species produced from one or more of the substrates, whereas the labelling on the left indicates the sizes of the three substrates. (**B**) The effect of blocking oligonucleotides on cleavage. As for (A), the substrates are shown schematically at the top of this panel. Closed black boxes indicate the binding sites to two oligonucleotides complementary to single-stranded segments in the intergenic region between *argX* and *hisR*. Their precise locations are shown in Supplementary Figure S4. Enzyme and initial substrate concentration and product staining (both panels) as Figure 2.

To investigate the substrate requirements for cleavage at E1, we deleted segments upstream of the 5′-end of *argX*, downstream of the 3′- end *leuT* and downstream of the 3′- end of *hisR* (Figure 4, panel A). Only deletion of the segment upstream of *argX* affected RNase E cleavage at E1. Although cleavage in this case was detected, the efficiency was reduced significantly by ∼8-fold. Thus, for all three sites, direct-entry cleavage is strongly influenced by an unpaired region that is adjacent, but not contiguous. Interestingly, the binding of oligonucleotides complementary to the single-stranded region in the intergenic region upstream of E1 increased the efficiency of cleavage by ∼3-fold in the absence of the 5′ leader region (Figure 4, panel B), but not in its presence (data not shown). We suggest that the oligonucleotide blocks a binding event that is ‘off path’ with regard to cleavage at E1. RNase E can bind many more sites than it cleaves efficiently (55) (our unpublished results). Regardless of the actual explanation, the effect of the complementary oligonucleotides on E1 cleavage is further evidence that single-stranded regions in addition to the segment in which cleavage occurs can influence the efficiency of cleavage.

**Figure 5.**
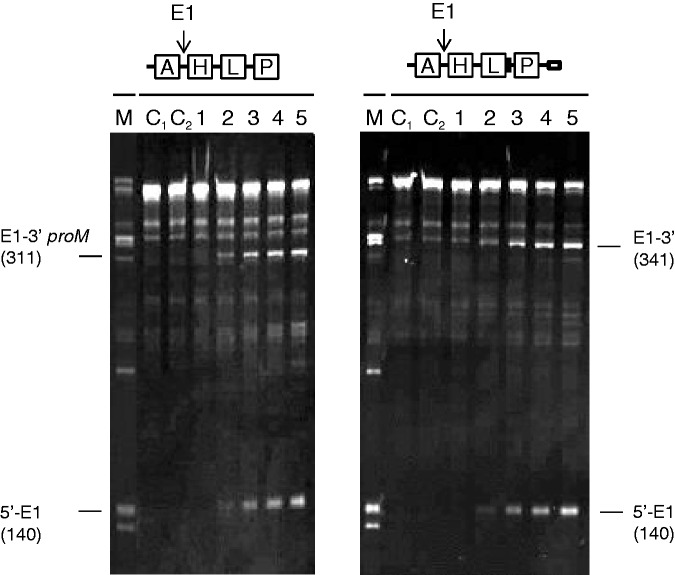
The requirement for adjacent single-stranded segments. The 5′-triphosphorylated substrates incubated with T170V and the positions of the resulting cleavages are shown schematically at the top of the panel. The first lacked the 3′ trailer, whereas the second had a complementary oligonucleotide overlapping the E3 site (see Supplementary Figure S4). As Figure 2, lanes 1–5 contain samples taken 0, 5, 15, 30 and 60 min after mixing substrate and enzyme, whereas lanes C_1_ and C_2_ correspond to substrate incubated without enzyme for 0 and 60 min. Lane M contains the products of cleaving full-length precursor with T170V. These are provided as size markers. Enzyme and initial substrate concentration and product staining as Figure 1.

Having found that an adjacent single-stranded segment(s) also regulated direct entry at E1, we tested whether these segments, or any other in the 5′ half of the precursor, could restore cleavage at E3 in the absence of the 3′ trailer, or cleavage at E5 in the absence of access to the single-stranded region encompassing the E3 site. The answer was negative (Figure 5). Within the context of the full-length transcript, we found that cleavage at E5 was still blocked using the E4 complementary oligonucleotide and that cleavage at E1 was still reduced by deletion of the 5′ leader (data not shown). Thus, single-stranded regions appear to be able to mediate direct entry at adjacent, but not distal sites. This may reflect the need for a particular local conformation to mediate efficient direct entry.

### The 5′-monophosphate-dependent cleavages

**Figure 6.**
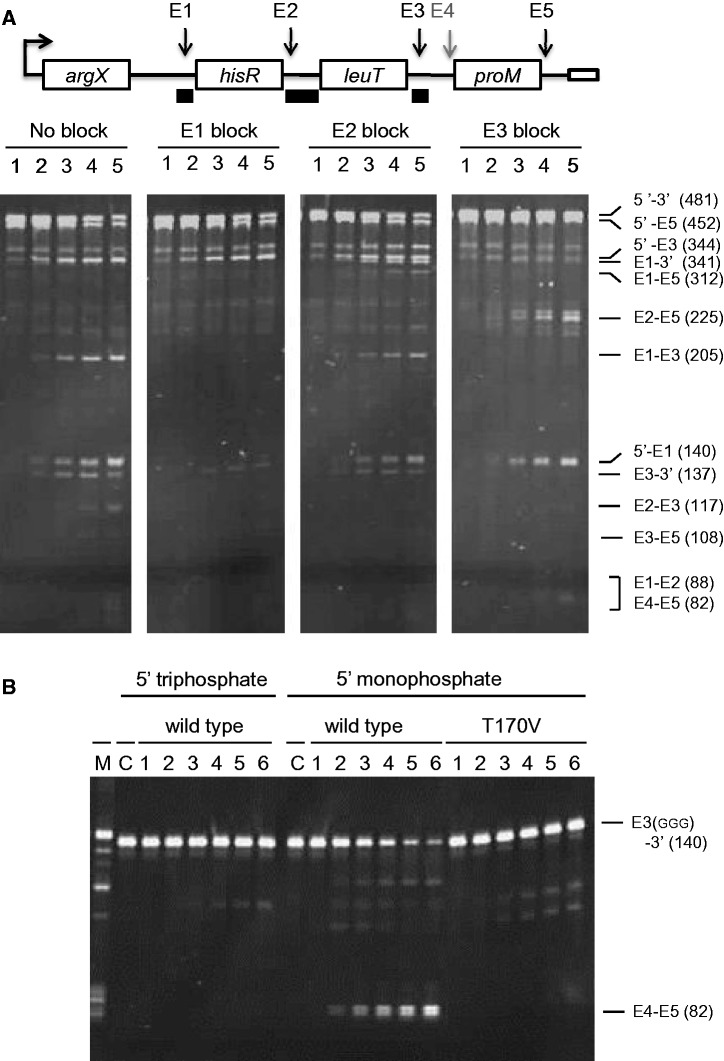
The 5′-monophosphate-dependent cleavages within the polycistronic *argX-hisR-leuT-proM* precursor. (**A**) Full-length precursors without or with a complementary oligonucleotide bound to a segment encompassing the E1, E2 or E3 sites were incubated with wild-type enzyme. Lanes 1–5 contain samples taken 0, 5, 15, 30 and 60 min after mixing substrate and enzyme. The labelling on the right indicates species produced from one or more of the substrates (see text for details). Enzyme and initial substrate concentration and product staining as Figure 1. (**B**) The E3 to 3′ intermediate was generated by *in vitro* transcription, and the 5′-monophosphorylated version was generated using TAP, as Figure 1. Labelling and numbering are also as Figure 1, except lane C contains substrate incubated without enzyme for 120 min. Enzyme and initial substrate concentrations and product staining as Figure 2. The E3 to 3′ intermediate generated by *in vitro* transcription migrated slightly slower than that generated by RNase E cleavage due to the presence of three extra G′s at the 5′ end: a requirement for efficient transcription. The position of E3(GGG) to 3′ and the E4 to E5 product are indicated on the left. The intermediates that are barely detectable correspond to E4 to 3′, E3(GGG) to E5 and trimming of the 5′ GGG nucleotides.

As indicated earlier in the text, a short intermediate accumulated during incubation with wild-type NTH-RNase E, but not its T170V equivalent (Figure 1). This intermediate corresponds to E2–E3 (117 nt). Moreover, the accumulation of E2–E3 in reactions with NTH-RNase E can be blocked by the binding of an oligonucleotide complementary to E1 (Figure 6, panel A). This suggested that cleavage at E2 is enabled by the 5′-monophosphorylated end produced by cleavage at E1. Consistent with this notion, the binding of an oligonucleotide complementary to the E2 site resulted in the accumulation of E1 to 3′ and E1 to E5. The binding of an oligonucleotide complementary to E1 or E2 also prevented the detection of the E3–E5 intermediate, which only ever accumulates to low levels, without affecting the levels of 5′ to E5, 5′ to E3 and E3 to 3′. This suggests that cleavage at E2 normally results in rapid 5′ monophosphate-stimulated cleavage at E3, in addition to the cleavage that occurs at this site via direct entry. Consistent with this model, the binding of an oligonucleotide complementary to the E3 site resulted in the accumulation of E2–E5. Furthermore, we also showed in an additional experiment that the generation of E4–E5 is stimulated by the 5′-monophosphorylated end generated by cleavage at E3. E4–E5 was generated efficiently from E3 to 3′, which was synthesized by *in vitro* transcription, provided the 5′-end was monophosphorylated and RNase E was not impaired in 5′ sensing (Figure 6, panel B). Thus, cleavage at E1 by direct entry appears to facilitate a series of 5′-monophosphate-dependent cleavages.

### Direct entry occurs in other tRNA precursors and is not limited by catalytic activity

**Figure 7.**
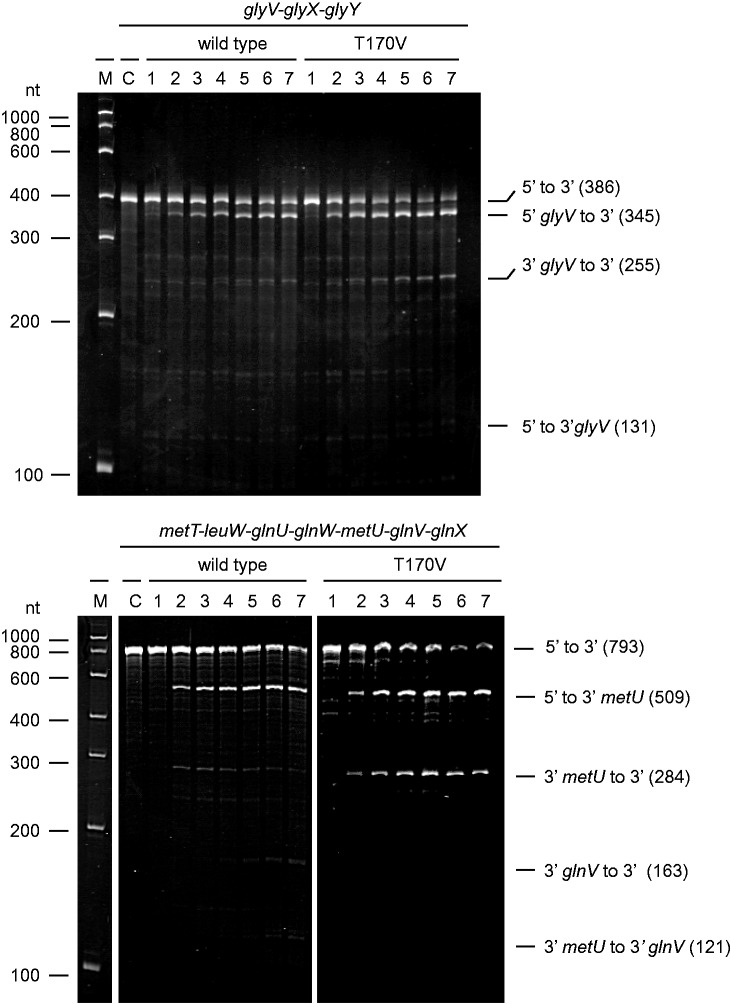
Initial cleavage of polycistronic *metT-leuW-glnUW-metU-glnVX* and *glyVXY* by 5′-monophosphate-independent mechanism. Both of the 5′-triphosphorylated precursors were generated by *in vitro* transcription and incubated with NTH-RNase E wild-type or T170V as indicated. Lanes 1–7 contain samples taken 0, 5, 15, 30, 60, 120 and 180 min after mixing substrate and enzyme, whereas lane C corresponds to substrate incubated without enzyme for 180 min. Lane M contains an RNA marker and the sizes (in nucleotides) are indicated on the left. Labelling on the right of each image indicates the positions of substrate and readily detectable products, which have been mapped tentatively according to size and positions of RNase E cleavages mapped by ourselves (our unpublished RNA-seq data). Enzyme and initial substrate concentration and product staining are as Figure 1.

**Figure 8.**
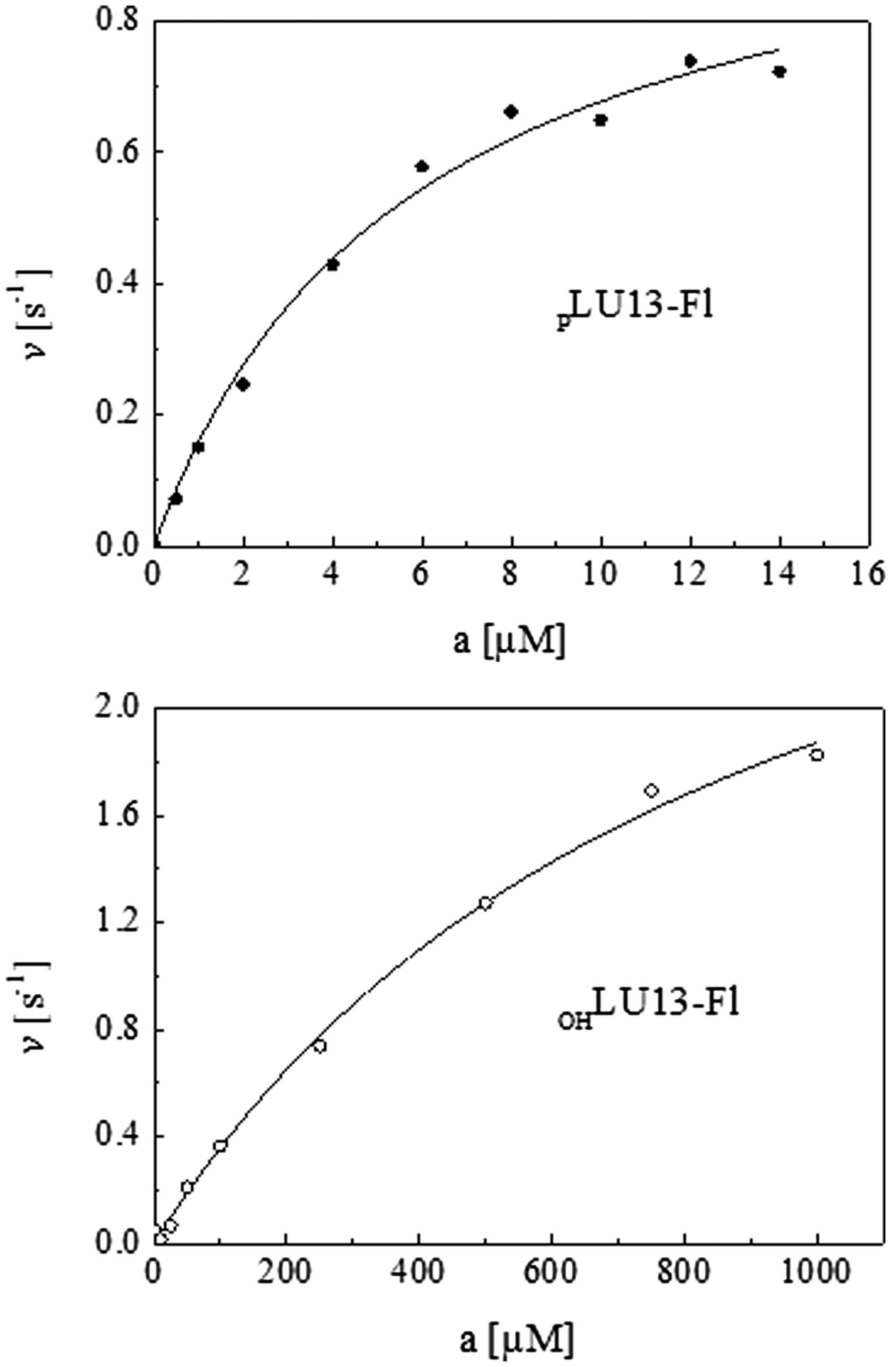
Michaelis–Menten analysis of the cleavage of a 5′-hydroxylated derivative of BR13. The reaction conditions and the measurements of initial rates were as described previously (21). The substrate was a 3′ fluorescein-labelled version of BR13 (56), referred to as LU13, that had GAG, rather than GGG, at the 5′-end (46). The cleavage of the 5′-monophosphorylated equivalent of the substrate was included as a control. The concentration of NTH-RNase E was 1 nM and the substrate was assayed over a concentration range of 500 nM to 14 µM and 10 µM to 1 mM for _P_LU13-Fl and _OH_LU13-Fl, respectively. Data are plotted as the initial rate (v) over substrate concentration (a). The values of *K*_M_ and *k*_cat_ were calculated from the curve of best fit to the Michaelis–Menten equation.

Although 5′-monophosphate-dependent cleavages have a role in *argX-hisR-leuT-proM* processing, the initial cleavage of this precursor at E1, E3 or E5 occurs via direct entry. The rate at which the full-length precursor diminished was largely independent of its 5′-phosphorylation status and a fully functional 5′-monophosphate-binding pocket in RNase E. Moreover, this does not appear to be specific to this particular tRNA precursor, as we found that 5′-triphosphorylated forms of polycistronic *metT-leuW-glnUW-metU-glnVX* and *glyVXY* precursors are also cleaved efficiently by RNase E T170V *in vitro* (Figure 7). Thus, direct entry appears to have a wide-spread role in tRNA processing. However, somewhat at odds with this notion was a report based on Michaelis–Menten analysis that the turnover number (value of *k*_cat_) is an order of magnitude lower in the absence of a 5′ monophosphate (32). Therefore, we decided to reinvestigate using high substrate concentrations (micro to millimolar) to minimize the extrapolation required to estimate the turnover number (Figure 8). Our analysis revealed that if anything the *k*_cat_ is slightly higher in the absence of a 5′ monophosphate. For the 5′-monophosphorylated oligonucleotide substrate, we obtained values of *K*_M_ and *k*_cat_ of 5.7 μM and 1.1 s^−1^, respectively, in good agreement with values obtained previously by us (24), whereas for the 5′-hydroxylated equivalent, we obtained *K*_M_ and *k*_cat_ of 0.9 mM and 3.5 s^−1^, respectively. Using these values, the efficiency of cleavage (*k*_cat_/*K*_M_) of the 5′-monophosphorylated substrate is calculated to be 50-fold higher than its 5′-hydroxylated equivalent. This matches well with the fold differences in cleavage efficiencies obtained previously for these substrates under non-saturating enzyme conditions (46). Thus, efficient cleavage does not require activation of the catalytic step by a 5′-monophosphorylated end.

## DISCUSSION

As an adequate pool of tRNAs for translation is absolutely essential for rapid bacterial growth (57,58), the maturation of tRNAs from their precursors is a key aspect of RNA metabolism. The initiation of tRNA maturation in *E. coli* is mediated by RNase E (6,7,59), which is renowned for being 5′-end dependent (1,2). However, the biochemical analyses described here, which used the previously characterized T170V mutant of RNase E (46) and the enzymatic manipulation of the 5′-phosphorylation status of transcripts, show that the initiation of the maturation of tRNAs encoded by the *argX-hisR-leuT-proM* precursor is not critically dependent on 5′-monophosphate-sensing (Figures 1–5), although it does have a role (Figure 6). Our work supports strongly the conclusion, based on the lack of accumulation of tRNA precursors in an *E. coli* strain containing a 5′-sensor mutant of RNase E, that the initiation of tRNA maturation, at least in some examples, is mediated by the direct entry of RNase E (49).

Our finding that direct entry requires access to single-stranded segments that are adjacent but not contiguous with single-stranded sites in which cleavage occurs fits with a model in which the simultaneous interaction of two single-stranded segments with RNase E can negate the requirement for a 5′-monophosphate group (46). The antiparallel arrangement of segments 5′ and 3′ to folded tRNA mirrors the antiparallel arrangement of the two RNA-binding channels in a principal dimer of RNase E (26). Thus, as found for other multimeric regulators (e.g. many bacterial transcription factors), simple cooperativity may be central to the initiation of tRNA processing by RNase E in *E. coli.* In addition to increasing the affinity of the interactions, cooperativity may also increase the selectivity. Despite having relatively low sequence specificity (53,54,60), RNase E cleaved the *argX-hisR-leuT-proM* precursor at only a limited number of sites (Figure 1). The molecular details of direct entry are probably best addressed by structural analysis of RNase E bound to a tRNA precursor or other direct-entry substrates. At this point, we do not exclude the possibility that another feature of tRNA precursors contributes to direct the entry of RNase E. We have preliminary evidence that tRNA has a role, perhaps in aligning the intergenic single-stranded regions optimally for efficient cleavage (unpublished data). The conformational context of sites cleaved by RNase E is well documented as having a role in controlling cleavage efficiency (61).

The initiation of tRNA processing by direct entry, which we show is not limited to the *argX-hisR-leuT-proM* precursor in *E. coli* (Figure 7), may extend to other bacteria. An analysis of the 3′ trailer sequences of tRNAs has found that AU-rich segments, which are recognizable by RNase E (53–55), are selectively conserved in bacteria with homologues of RNase E (48). Moreover, a preliminary analysis of transcripts in the *E. coli* transcriptome that are cleaved efficiently by T170V *in vitro* (our unpublished data) suggests that direct entry may also be a common feature of mRNA degradation, as proposed previously (46). By characterizing and comparing additional substrates, it should be possible to determine the extent to which the conformational context of single-stranded segments places limits on direct entry; some initial cleavages by RNase E in *E. coli* are clearly dependent on the generation of a 5′-monophosphorylated end (36). Furthermore, evidence has emerged recently that the decay of a regulatory RNA requires the physical recruitment of RNase E via an adaptor protein (62).

**Figure 9.**
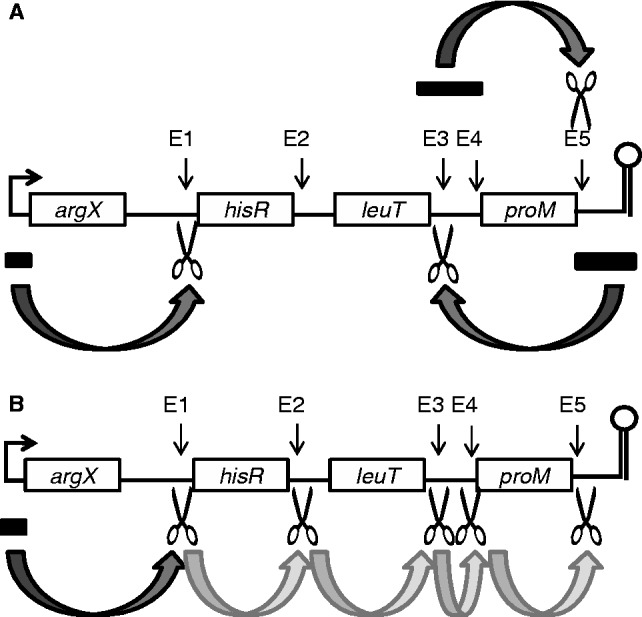
Summary of RNase E direct entry and 5′-monophosphate-dependent cleavages in the *argX-hisR-leuT-proM* precursor. (**A**) The requirement of adjacent but not contiguous single-stranded segments for direct-entry cleavage. Closed rectangles indicate sites to which RNase E binds as part of cooperative interactions that mediate direct entry; the dark gray arrows indicate a requirement of binding at the indicated region for efficient cleavage at the site shown by a pair of scissors. Efficient direct-entry cleavage at E1 requires binding to the 5′ leader. Interaction with the E3–E4 region leads to efficient cleavage at E5 and conversely binding to the E5 region results in efficient cleavage at E3. Direct-entry cleavage at E3 and E5 are mutually exclusive. (**B**) 5′-monophosphate dependent cleavages within the *argX-hisR-leuT-proM* precursor. Binding and cleavage by direct entry are labelled as panel (A). Direct-entry cleavage at E1 results in the generation of a 5′-monophosphate, which in turn enables a series of 5′-monophosphate-dependent cleavages, represented by the pale gray arrows, starting at E2 and followed by E3, and then E4 and E5. The final products of RNase E cleavage of the *argX* precursor may be produced by a combination of direct entry and 5′-end-dependent cleavage.

In addition to shedding light on the role of direct entry, our study also provides an example, perhaps the clearest to date, that the generation of a 5′ monophosphate as the result of an initial cleavage can trigger multiple cleavages (Figure 6), as was suggested when RNase E was first found to be able to interact with 5′-monophosphorylated ends (34). Cleavage at E1 was followed by cleavage at E2, then E3 and finally E4 and E5. Thus, although processing *in vivo* might be initiated by direct entry, subsequent steps can be mediated by 5′-end-dependent cleavages (Figure 9). The location of the 5′-monophosphate binding pocket next to the active site is ideal to engage the 5′-end of the downstream product of cleavage. Engagement with this pocket appears, from the Michaelis–Menten analysis reported here (Figure 8), to enhance primarily the affinity of the overall interaction, as found previously for *E. coli* RNase G (21).

Finally, we would point out that the absence of detectable intermediates of tRNA processing in a 5′-sensor mutant strain of *E. coli* (49) is not at odds with a role for 5′-end-dependent cleavages. It simply indicates that these cleavages are not critical. For example, it is possible that some tRNAs can be separated endonucleolytically by RNase P, which generates the mature 5′-end of all tRNAs (47), in the absence of a 5′-end-dependent cleavage that would normally occur upstream within the same intergenic region. The 3′ tails of tRNAs separated from the precursor by RNase P cleavage would then be trimmed 3′ exonucleolytically *in vivo*, as found for 3′ tails generated by RNase E cleavage (47). A role for RNase P in the separation of tRNAs has been documented for the *metT-leuW-glnUW-metU-glnVX* precursor. In accordance with our biochemical analysis of this transcript (Figure 7), the only sites of RNase E cleavage detected *in vivo* mapped downstream of *metU* (63).

## SUPPLEMENTARY DATA

Supplementary Data are available at NAR Online.

## FUNDING

Funding for open access charge: Biotechnology and Biological Sciences Research Council [BB/I001751/1] grant and studentship (to K.J.M.).

*Conflict of interest statement*. None declared.

## Supplementary Material

Supplementary Data
