# Supplementary material for: Adjacent single-stranded regions mediate processing of tRNA precursors by RNase E direct entry
Source: Nucleic Acids Res. 2014 Jan 21;42(7):4577–89. doi: 10.1093/nar/gkt1403 (PMC3985628; doi:10.1093/nar/gkt1403)
Supplement: Supplementary Data [file supp_42_7_4577__index.html]

Adjacent single-stranded regions mediate processing of tRNA precursors by RNase E direct entry — Adjacent single-stranded regions mediate processing of tRNA precursors by RNase E direct entry — Supplementary Data 

# Adjacent single-stranded regions mediate processing of tRNA precursors by RNase E direct entry

## Supplementary Data

files

**Files in this Data Supplement:**

- Supplementary Data - pdf file
