# Supplementary material for: Adjacent single-stranded regions mediate processing of tRNA precursors by RNase E direct entry
Source: Nucleic Acids Res. 2014 Jan 21;42(7):4577–89. doi: 10.1093/nar/gkt1403 (PMC3985628; doi:10.1093/nar/gkt1403)
Supplement: Supplementary Data [file supp_gkt1403_nar-03176-z-2013-File003.pdf]

Figure S1

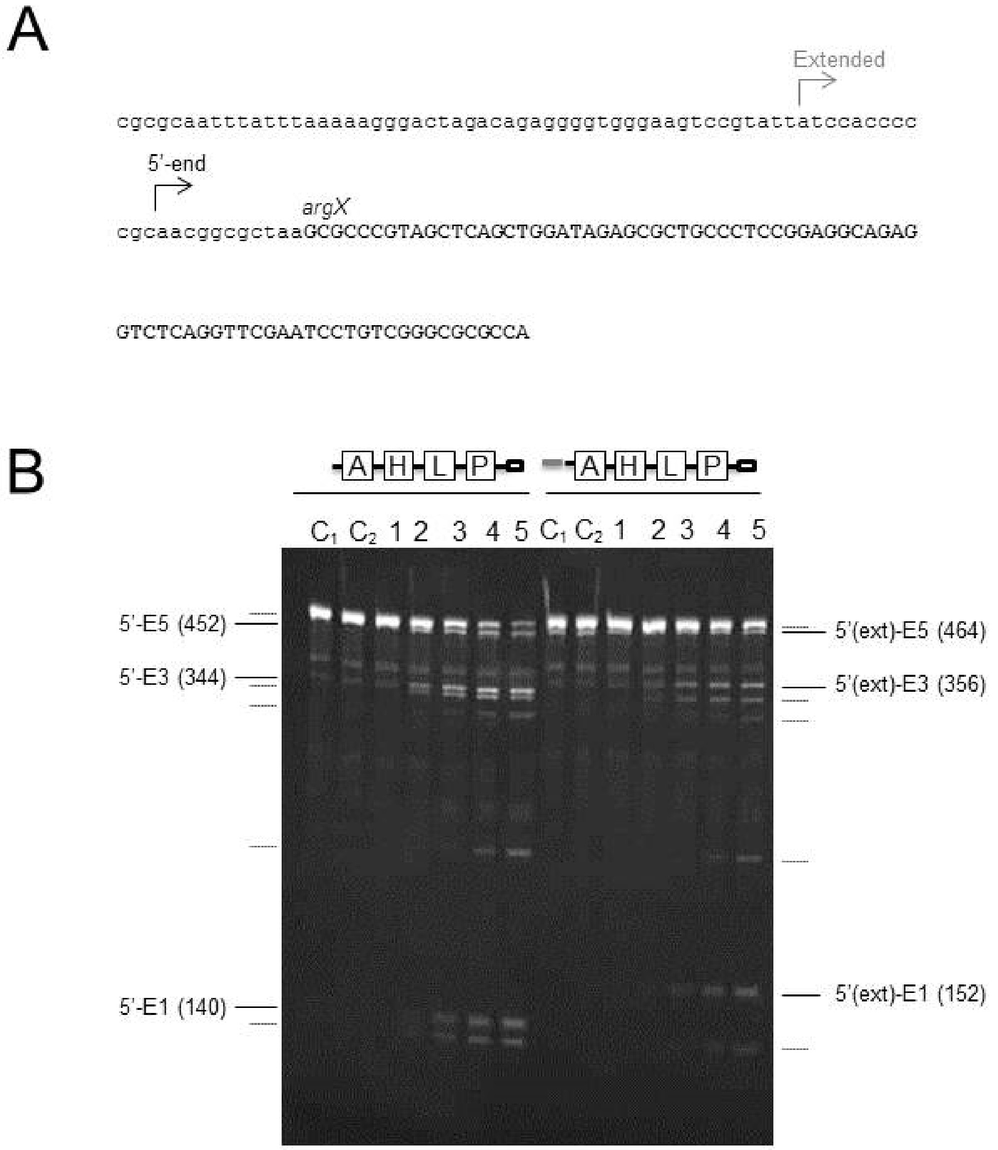

Figure S1. Identifying cleavage products corresponding to the 5' end of the *argX-hisR-leuT-proM* precursor **(A)** Annotated sequence of the 5'-extended precursor. The start sites of the original and extended substrates are indicated by black and grey arrows respectively. The forward primer used to generate the extended PCR product for transcription was 5'-ATCCTAATACGACTCACTATAGGGATCCACCCCCGCAAC, where the T7 polymerase promoter is underlined. **(B)** The 5' extended form of the *argX* precursor was generated by *in vitro* transcription and the 5'-triphosphorylated transcripts were incubated with T170V and the products analysed by denaturing gel electrophoresis. The enzyme and initial substrate concentration at the start of each reaction were 7 nM and 250 nM, respectively. The RNA was stained using ethidium bromide. Lanes 1-5 contain samples taken at 0, 5 15, 30 and 60 min following mixing of substrate and enzyme, while lanes C_1 and C_2 correspond to substrate incubated without enzyme for 0 and 60 min, respectively. Products with the 5'-end of the original transcript are labelled on the left and the corresponding products with an additional 12 nucleotides at the 5'-end are labelled on the right.

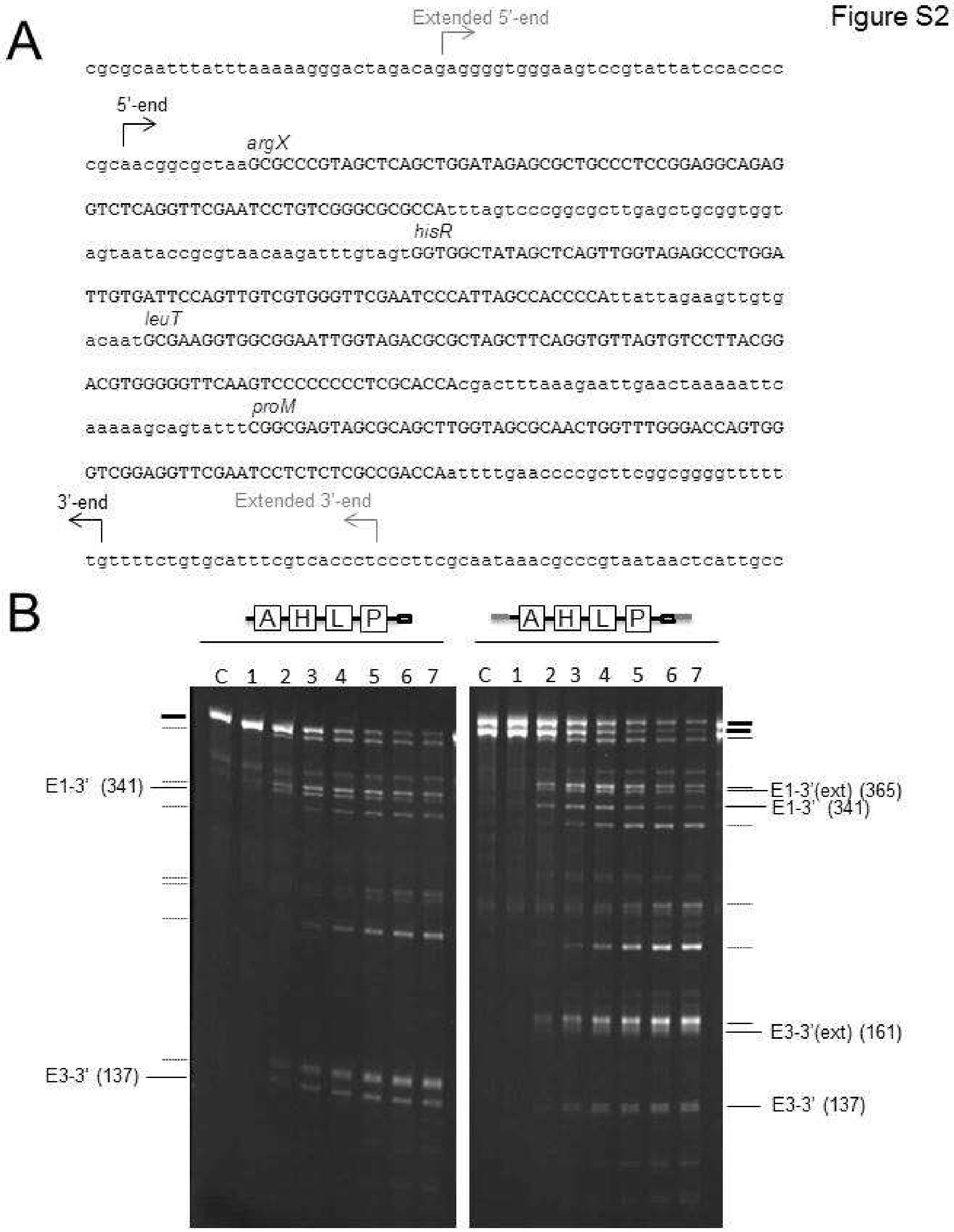

Figure S2. Identifying cleavage products corresponding to the 3' end of the *argX-hisR-leuT-proM* precursor. **(A)** Annotated sequence of the 3'-extended precursor, which was also extended at the 5' end. The start and termination sites of the original and extended precursor are indicated by black and grey arrows, respectively. The extended precursor in this analysis had an extra 32 nucleotides at the 5' end and 24 nucleotides at the 3' end. The sequences of the forward and reverse primers used to generate the extended PCR product for transcription were 5'-CGCGTAATACGACTCACTATAGGGAGGGGTGGGAAGTCCGT ATTA and 5'-AGGGTGACGAAATGCACAGAA, respectively. The T7 polymerase promoter in the forward primer is underlined. **(B)** The extended precursor was generated and analysed as described in Figure S1. Products with the 3'-end of the original transcript are labelled on the left and the corresponding products with an additional 24 nucleotides at the 3' end are labelled on the right. Short, solid black bars on the right indicate the positions of products corresponding to the 5' end.

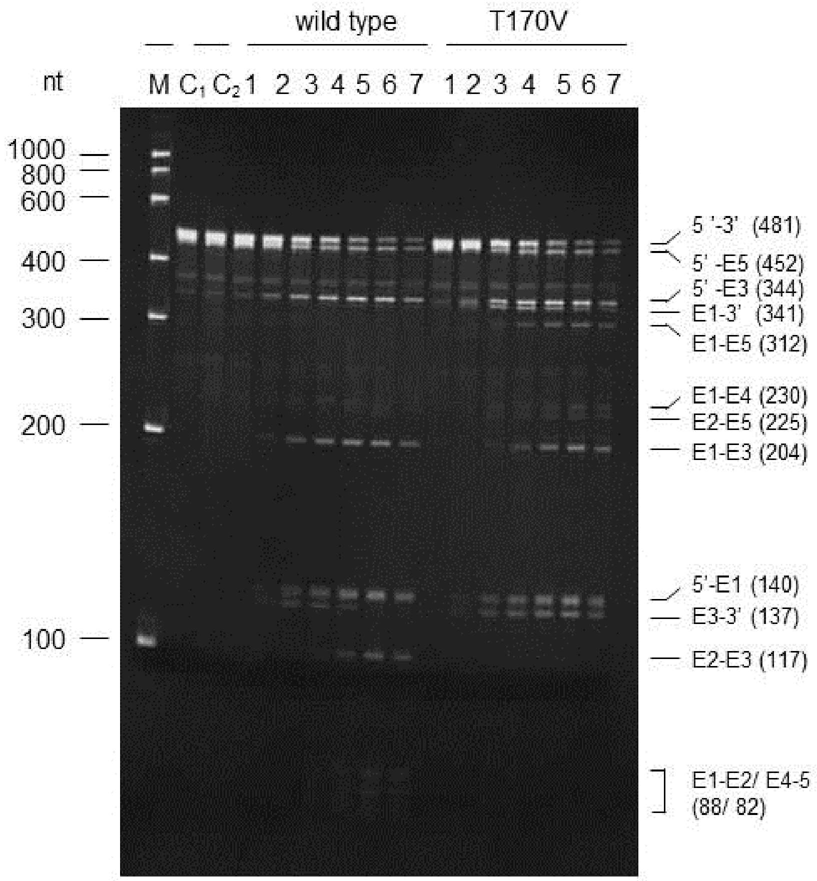

Figure S3. RNase E cleavage of the polycistronic *argX-hisR-leuT-proM* precursor electrophoresed against an RNA ladder. The triphosphorylated precursor was incubated with wild-type and T170V NTH-RNase E. Enzyme and initial substrate concentrations at the start of the reaction were 5 nM and 180 nM, respectively. Lanes 1-7 contain samples taken 0, 5, 15, 30, 60 120 and 180 min after mixing substrate and enzyme. Lanes C_1 and C_2 contain substrate incubated without enzyme for 0 and 180 min, respectively. The identities of the bands are labelled on the right of the panel. Lane M contains an RNA marker and the sizes (in nucleotides) are indicated on the left.

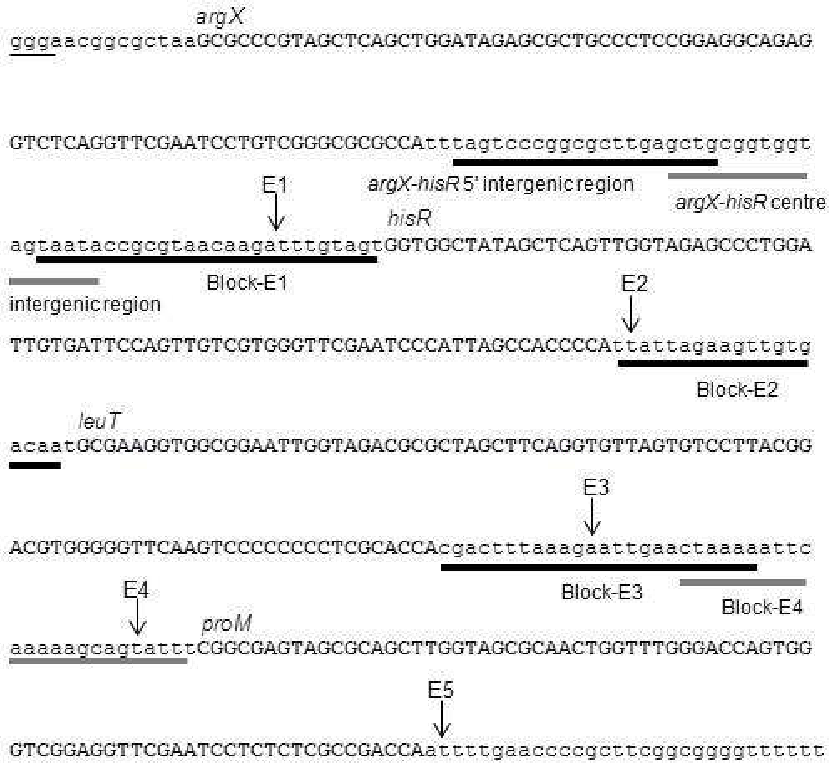

Figure S4. Annotated sequence of the *argX-hisR-leuT-proM* precursor. The triple G generated from *in vitro* transcription of the PCR template is underlined. The tRNA genes are capitalised and the intergenic regions are shown in lower case. The positions of RNase E cleavages are indicated above the sequence by arrows (6)(our unpubl. data). The sequences blocked by complementary oligonucleotides are indicated below the sequence by thick black or grey lines.

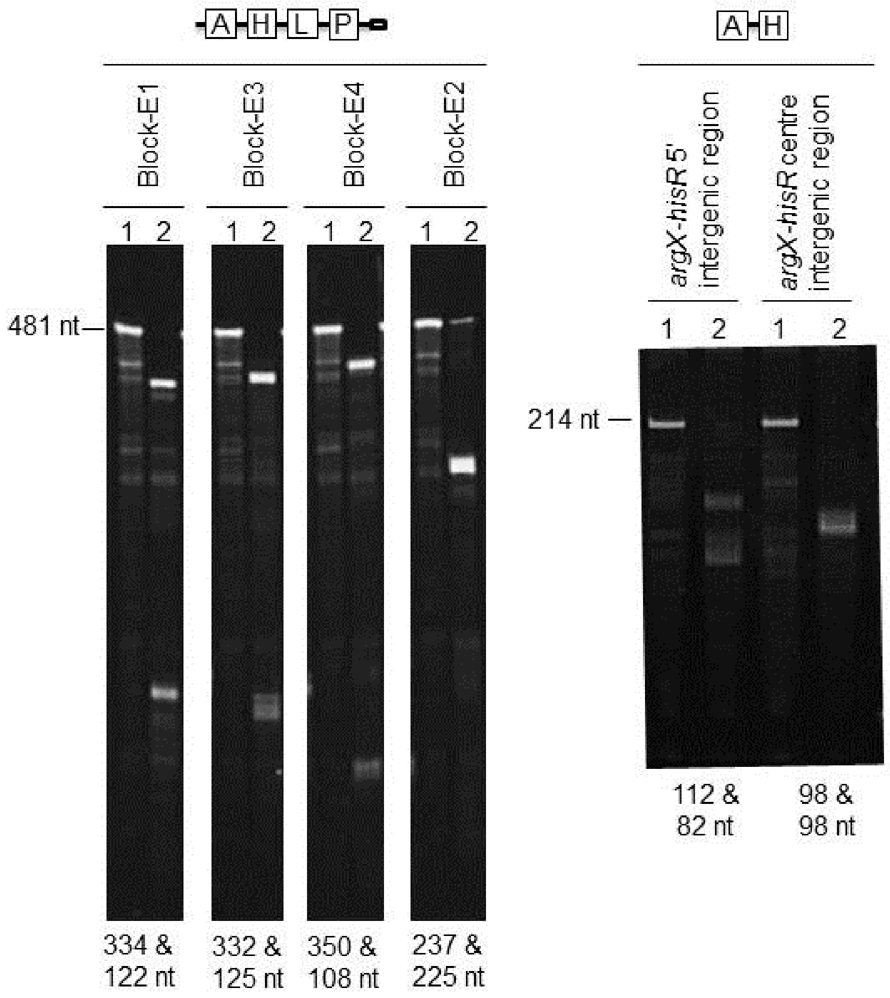

Figure S5. Annealing of complementary oligonucleotides to the *argX-hisR-leuT-proM* precursor. Oligonucleotides were annealed to complementary sequences within the transcript, followed by RNase H digestion as described in Materials and Methods. The RNA was stained using SYBR® Gold stain (Life Technologies). The transcript and complementary oligonucleotide combinations are indicated at the top of the gels. The annealed RNA: oligonucleotide was incubated in the absence (lane 1) or presence (lane 2) of RNase H. Expected sizes of the products of RNase H digestion, in nucleotides, are indicated at the bottom of each panel.
